# Supplementary material for: Tailless/TLX reverts intermediate neural progenitors to stem cells driving tumourigenesis via repression of asense/ASCL1
Source: eLife. 2020 Feb 19;9:e53377. doi: 10.7554/eLife.53377 (PMC7058384; doi:10.7554/eLife.53377)
Supplement: Supplementary file 1. [file elife-53377-supp1.docx]

| Figure | GAL4 line | Crossed to | Temperature |
| --- | --- | --- | --- |
| 1C  1D-D’ | *yw*,UAS*-mCD8-GFP;+; pntP1*-GAL4 | n/a | 25 ˚C throughout |
| 2A-B’’ | *w; Ay*-GAL4,UAS-*GFP*; *pntP1*-GAL4 | Control: *w*; +; UAS-*FLP*  *tll*-miRNA[s]: *w*; UAS-*tll*-miRNA[s]; UAS-*FLP* | 25 ˚C until larval hatching, 30 hours at 29 ˚C |
| 3B-B’ | *w*; *Ay*-GAL4,UAS-*GFP*/(CyO*act*-GFP); *erm*-GAL4, *tub*-GAL80^ts^ | Control: *w*; UAS-*FLP*/CyO*act*-GFP; +  Tll OE: *w*; UAS-*FLP*/CyO*act*-GFP; UAS-*tll*/TM6B | 18 ˚C until larval hatching, 3 days at 29 ˚C |
| 3D-D’ | *w*; *+; GMR71C09*-GAL4*,* UAS-*mCD8-GFP* | Control: *w^1118^*; +; +  Tll OE: *w*; +; UAS-*tll*/(TM6B) | 25 ˚C throughout |
| 3E-E’ | *w*; FRTG13,*vGlut^OK371^*-GAL4,UAS-*mCD8-GFP*/CyO*act*-GFP; + | Control: *w^1118^*; +; +  Tll OE: *w*; +; UAS-*tll*/(TM6B) | 25 ˚C throughout |
| 4A-A’ | *w; wor*-GAL4,UAS-mCD8-GFP; *tub*-GAL80^ts^ | Control: *w^1118^*; +; +  Tll OE: *w*; +; UAS-*tll*/(TM6B) | 18 ˚C until mid third instar, 29 ˚C until eclosion |
| 4B-B’  4C-C’  4D-D’ | *w; wor*-GAL4,UAS-mCD8-GFP; *tub*-GAL80^ts^ | Control: *w^1118^*; +; +  Tll OE: *w*; +; UAS-*tll*/(TM6B) | 18 ˚C until larval hatching, 3 days at 29 ˚C |
| 5A-A’’ | *w*; + ; *erm*-GAL4 | Control: *w*; UAS-*RedStinger*,UAS-*FLP*,*Ubi*-p63E,FRT-STOP-FRT *Stinger*/(CyO*act*-GFP)*; +*  Tll OE: *w*; UAS-*RedStinger*,UAS-*FLP*,*Ubi*-p63E,FRT-STOP-FRT *Stinger*/(CyO*act-*GFP)*; UAS-tll*/(TM6B)  Human TLX OE: *w*; UAS-TLX/(CyO*act*-GFP); UAS-*RedStinger*,UAS-*FLP*,*Ubi*-p63E,FRT-STOP-FRT *Stinger/*(TM6B) | 25 ˚C throughout |
| 6A-A’’  6B-B’’ | *w; wor*-GAL4,UAS-mCD8-GFP; *tub*-GAL80^ts^ | Control: *w*; UAS-*lacZ*; UAS-*mCD8-GFP*  Tll OE: *w*; UAS-*lacZ*/(CyO*act*-GFP); UAS-*tll*/(TM6B)  Ase Rescue: *w*; UAS-*ase*/(CyO*act*-GFP); UAS-*tll*/(TM6B) | 18 ˚C until larval hatching, 3 days at 29 ˚C |
| Figure 1 – figure supplement 1A-C | *w*; +; *pntP1*-GAL4 | *w*; *tll-EGFP*,UAS-*myr-mRFP*/(CyO*act*-GFP); + | 25 ˚C throughout |
| Figure 2 – figure supplement 1B-B’ | *w; wor*-GAL4,UAS-mCD8-GFP; *tub*-GAL80^ts^ | Control: *w^1118^*; +; +  *tll*-miRNA[s]: *w*; UAS-*tll*-miRNA[s]; + | 18 ˚C until larval hatching, 3 days at 29 ˚C |
| Figure 2 – figure supplement 1C-C’ | *w; wor*-GAL4,UAS-mCD8-GFP; *tub*-GAL80^ts^ | Control: *w^1118^*; +; +  *tll*-shRNA: w; *tll*-shRNA; + | 18 ˚C until larval hatching, 3 days at 29 ˚C |
| Figure 2 – figure supplement 1D-D’, E, F | *hsFLP^122^*; *wor*-GAL4, UAS-*mCD8-mCherry*/(CyO*act*-GFP); FRT82B, *tub*-GAL80 | Control clones: *w*; *erm*-*lacZ*; FRT82B  *tll* null clones: *w*; *erm*-*lacZ*; FRT82B, *tll^l49^*/TM6B. | 25 ˚C throughout.  Heatshock 24 hours ALH (5 minutes 37 °C, 5 minutes rest at room temperature, 1 hour 37 °C). Larvae were dissected 72 hours later |
| Figure 2 – figure supplement 1G | *w*; *insc*-GAL4; *tub*-GAL80^ts^ | Control: *w^1118^*; +; +  *tll*-miRNA[s]: *w*; UAS-*tll*-miRNA[s]; + | 18 ˚C until larval hatching, 3 days at 29 ˚C |
| Figure 2 – figure supplement 2B and D | *w; Ay*-GAL4,UAS-*GFP*; *pntP1*-GAL4,*erm-CD4-tdTomato* | Control: *w*; +; UAS-*FLP*  *tll*-miRNA[s]: *w*; UAS-*tll*-miRNA[s]; UAS-*FLP* | 25 ˚C until larval hatching, 19 hours at 29 ˚C |
| Figure 2 – figure supplement 2C-C’ | *w; Ay*-GAL4,UAS-*lacZ(nls),pnt-GFP*/CyO*act*-GFP; *pntP1*-GAL4 | Control: *w*; +; UAS-*FLP*  *tll*-miRNA[s]: *w*; UAS-*tll*-miRNA[s]; UAS-*FLP* | 25 ˚C until larval hatching, 30 hours at 29 ˚C |
| Figure 3 – figure supplement 1A | *w*; + ; *erm*-GAL4, UAS-*mCD8-GFP* | n/a | 25 ˚C throughout |
| Figure 3 – figure supplement 1C-C’ and D-D’ | *w*; *Ay*-GAL4,UAS-*GFP*/(CyO*act*-GFP); *erm*-GAL4, *tub*-GAL80^ts^ | Control: *w*; UAS-*FLP*/CyO*act*-GFP; +  Tll OE: *w*; UAS-*FLP*/CyO*act*-GFP; UAS-*tll*/TM6B | 18 ˚C until larval hatching, 3 days at 29 ˚C |
| Figure 4 – figure supplement 1A-A’ | *btd*-GAL4*,FRT19A/*FM7*act-GFP*; *+*; *tub*-GAL80^ts^ | Control: *w*; *erm*-*mCD8-GFP*; UAS-*myr-mRFP*/TM6B  Tll OE: *w*; *erm*-*mCD8-GFP*; UAS-*tll*, UAS*-myr-mRFP*/TM6B | 18 ˚C until larval hatching, 3 days at 29 ˚C |
| Figure 5 – figure supplement 2A-A’ | *w*; FRTG13,*vGlut^OK371^*-GAL4,UAS-*mCD8-GFP*/CyO*act*-GFP; + | Control: *w^1118^*; +; +  Human TLX: *w*; UAS-*TLX*; *+* | 25 ˚C throughout |
| Figure 6 – figure supplement 1B | *dpn*>KDRTs-stop-KDRTs>GAL4*; ase*-GAL80/CyO*act*-GFP; + | *w*; UAS-*mCD8-GFP*; *stg14*-KD | 25 ˚C throughout |
| Figure 6 – figure supplement 2A-A’’ | *w; wor*-GAL4,UAS-mCD8-GFP; *tub*-GAL80^ts^ | Control: *w*; UAS-*lacZ*; UAS-*mCD8-GFP*  Tll OE: *w*; UAS-*lacZ*/(CyO*act*-GFP); UAS-*tll*/(TM6B)  Ase Rescue: *w*; UAS-*ase*/(CyO*act*-GFP); UAS-*tll*/(TM6B) | 18 ˚C until larval hatching, 3 days at 29 ˚C |
| Figure 6 – figure supplement 2B-B’ | *w; Ay*-GAL4,UAS-*GFP*; *pntP1*-GAL4 | Control: *w*; +; UAS-*FLP*  Ase OE: *w*; UAS-*ase*; UAS-*FLP* | 25 ˚C throughout |

Supplementary Table 1: *Drosophila* genotypes and experimental conditions.
